# Delirium and incident nursing home admission among people with and without dementia

**DOI:** 10.1093/ageing/afag151

**Published:** 2026-05-30

**Authors:** Markus J Haapanen, David D Ward, Ding Ma, Emily H Gordon, Kenneth Rockwood, Ruth E Hubbard

**Affiliations:** Centre for Health Services Research, Faculty of Health, Medicine and Behavioural Sciences, The University of Queensland, Princess Alexandra Hospital, Woolloongabba, Queensland, Australia; Australian Frailty Network, The University of Queensland, Woolloongabba, Queensland, Australia; Department of General Practice and Primary Health Care, University of Helsinki, Helsinki, Finland; Centre for Health Services Research, Faculty of Health, Medicine and Behavioural Sciences, The University of Queensland, Princess Alexandra Hospital, Woolloongabba, Queensland, Australia; Australian Frailty Network, The University of Queensland, Woolloongabba, Queensland, Australia; Centre for Clinical Research, Faculty of Health, Medicine and Behavioural Sciences, The University of Queensland, Herston, Queensland, Australia; Centre for Health Services Research, Faculty of Health, Medicine and Behavioural Sciences, The University of Queensland, Princess Alexandra Hospital, Woolloongabba, Queensland, Australia; Australian Frailty Network, The University of Queensland, Woolloongabba, Queensland, Australia; Division of Geriatric Medicine, Faculty of Medicine, Dalhousie University, Halifax, Nova Scotia, Canada; Centre for Health Services Research, Faculty of Health, Medicine and Behavioural Sciences, The University of Queensland, Princess Alexandra Hospital, Woolloongabba, Queensland, Australia; Australian Frailty Network, The University of Queensland, Woolloongabba, Queensland, Australia

**Keywords:** delirium, dementia, nursing home admission, accelerated failure time models, competing risk, older people

## Abstract

**Background:**

Delirium is a common, preventable hospital complication and a recognised risk factor for cognitive decline, but its association with both the risk and timing of nursing home admission remains uncertain, particularly in relation to dementia.

**Methods:**

We conducted a matched cohort study of hospitalised UK Biobank participants with linked hospital records (1997–2022). Episodes of delirium were identified using ICD-10 codes and matched 1:1 to non-delirium control episodes within no dementia (*n* = 13 004) and dementia (*n* = 1790) groups. Matching was performed on age, sex, hospital frailty risk score, primary diagnosis, length of stay and intensive care admission. Incident nursing home admissions were identified from hospital discharge records. Fine–Gray models estimated adjusted subdistribution hazard ratios (SHRs), while accelerated failure-time models quantified time to admission.

**Results:**

Delirium was associated with a higher risk of nursing home admission in participants without dementia (SHR 1.23, 95% CI 1.14–1.34) and with pre-existing dementia (SHR 1.22, 95% CI 1.07–1.38). Delirium also precipitated admission by ~40% in both groups, corresponding to 1.6 years earlier (95% CI 1.1–1.9) at a survival probability of 0.90 in those without dementia and 1.3 years earlier (95% CI 0.6–1.8) at a survival probability of 0.70 in those with dementia.

**Conclusions:**

Hospital-recorded delirium was associated with both if and when older adults entered nursing home care. In contrast to previous evidence, this association was present regardless of pre-existing dementia. These findings highlight delirium prevention as a potential strategy to delay—or avert—transitions to long-term care.

## Key points

- In-hospital delirium was associated with both an increased risk of nursing home admission and earlier admission.
- The association between delirium and nursing home admission was similar in people with and without pre-existing dementia.
- Delirium precipitated nursing home admission by more than a year on average, highlighting its impact on the timing of admission.
- Among individuals without dementia, recurrent delirium showed a dose–response relationship with earlier nursing home admission.
- Findings may underestimate delirium burden due to reliance on hospital coding and limited generalisability of the cohort.

## Introduction

Admission to nursing home care is costly for individuals and health systems [1] and is associated with lower quality of life, social isolation and increased mortality [2–4]. Preventing or delaying admission is therefore a public health priority. Dementia remains one of the strongest predictors [5]—57% enter nursing home care within 5 years of diagnosis [6].

Delirium—an acute, preventable and potentially life-threatening disturbance of attention and cognition that affects roughly one in four hospital admissions [7–10]—is increasingly recognised as a modifiable risk factor for dementia [11–13]. Delirium may present on admission or arise as a complication during hospitalisation. In a study of general medical inpatients, 68% of cases were present at admission, 17% developed during admission and 15% were observed in both settings [14]. Ward-based multicomponent programmes can prevent up to 40% of incident delirium occurring during hospitalisation [8, 15], suggesting that delirium prevention could substantially reduce nursing home admissions. Still, evidence on delirium and subsequent nursing home admission is mixed: some studies show no association [16–18], whereas others report two to five times higher risk [19–28] and two to nine times higher risk among those with dementia [16, 19–21, 29]. Such variation arises from limitations such as small sample sizes, limited duration of follow-up (generally up to 24 months), inadequate consideration of mortality and unaccounted variation in individual and hospital episode characteristics. Furthermore, most studies present relative risks without translating them into practical terms—for example, how much earlier a person enters a nursing home after delirium compared to another person without delirium. As a result, clinicians and policymakers remain uncertain about the implications of delirium prevention, inhibiting the actions needed to make progress [30].

This study examines the associations between delirium and both the risk and timing of nursing home admission in individuals with and without dementia within the UK Biobank cohort. By matching patient and hospital episode characteristics, we aimed to quantify (i) the impact of delirium on the risk of nursing home admission and (ii) how much earlier admission occurs following delirium compared with no delirium, while also exploring a dose–response relationship with the number of delirium episodes.

## Materials and methods

We examined the impact of delirium on incident nursing home admissions using the UK Biobank, a prospective cohort of ~500 000 participants aged 40–69 years at recruitment, with linkage to hospital inpatient records from 1997 to 2022.

**Figure 1:**
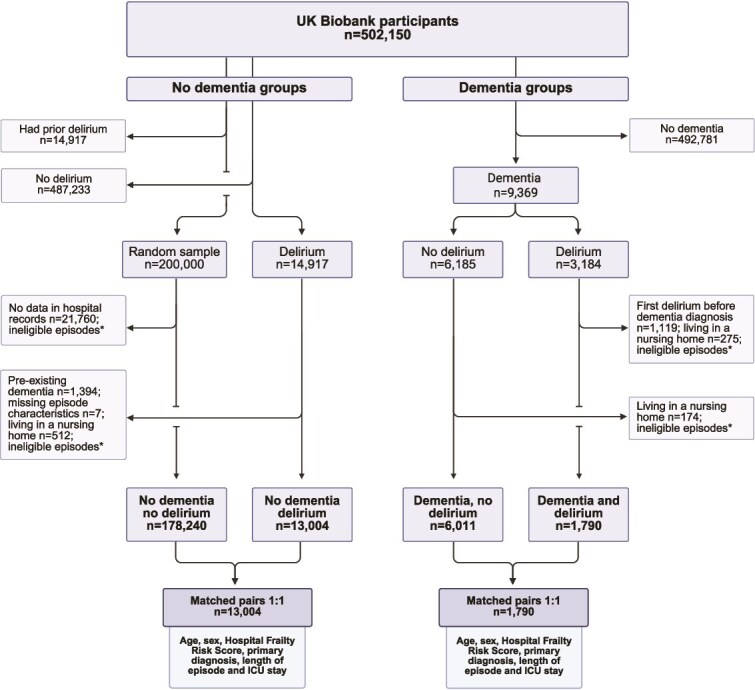
Flow diagram of participant selection and 1:1 matching for dementia and delirium cohorts in UK Biobank. ^*^Ineligible episodes refer to episodes following first nursing home admission (all groups) or first dementia diagnosis (no dementia groups).

We employed a matched cohort design among UK Biobank participants who had been hospitalised at least once (Figure 1). Participants with and without dementia were identified using ICD-10 codes from hospital records (Supplementary Table 1). Within both groups, episodes of delirium were similarly identified using ICD-10 codes (Supplementary Table 2); the first recorded episode was defined as the index episode. We excluded participants who had ever been admitted to a nursing home on or before their index episode date, as indicated by the hospital discharge variable. Additionally, in the dementia group, individuals with a history of delirium prior to their dementia diagnosis were excluded. For those with delirium, the total number of episodes was recorded; participants without any recorded episodes of delirium were classified into the ‘no delirium’ group.

The presence of dementia and occurrence of delirium permitted four categories of participants: (i) no dementia, without delirium (*n* = 487 233), (ii) no dementia with delirium (*n* = 13 004), (iii) dementia without delirium (*n* = 6011) and (iv) dementia with delirium (*n* = 1790).

### Matching procedure

We used 1:1 nearest-neighbour propensity score matching on prespecified clinically relevant covariates to make the delirium and non-delirium episodes more comparable on measured covariates. Within both the no dementia and dementia groups, each delirium index episode was matched 1:1 to a control episode with no record of delirium. In the no dementia group, 13 004 episodes of delirium were matched to control episodes randomly drawn from a large control pool—200 000 individuals (1.57 million episodes). In the dementia group, 1790 episodes of delirium were matched to episodes from 6011 controls (89 734 episodes). Matching was restricted to episodes during which participants were not residing in nursing homes and, for the no dementia group, before the onset of any recorded dementia.

Matching was based on patient and episode characteristics with potential to confound the association between delirium and incident nursing home admission, including age (years), sex (male or female), hospital frailty risk score [31] (continuous; calculated from episodes occurring within the 2 years preceding each index or control episode; for participants without any hospital admissions during this look-back period, the Hospital Frailty Risk Score (HFRS) was assigned a value of 0), the primary diagnosis, episode length of stay (days) and intensive care unit length of stay of the index episode. Sociodemographic covariates—household income and Townsend Deprivation Index—were deliberately omitted because they were measured years before admission; including them would have limited match accuracy on hospital episode characteristics and treating them instead as adjustment covariates allowed later sensitivity analyses of their independent influence. Matching was performed without replacement; each participant with delirium was matched to a unique participant without delirium.

### Covariates

Sociodemographic covariates included annual household income (categorised as <£18 000, £18 000–30 999, £31 000–51 999, £52 000–100 000, >£100 000 and ‘Do not know’/‘Prefer not to answer’) and the Townsend Deprivation Index derived from residential postcode at recruitment.

### Outcome

The primary outcome was time (in years) to first nursing home admission. Incident admissions were identified using the hospital discharge destination variable (DISDEST; Data coding 267). Episodes were classified as nursing home admissions where the discharge destination was recorded as nursing home, residential care home or group home. The discharge date of the first such episode was recorded as the date of nursing home admission. Dates of death were obtained from linked mortality records.

### Statistical analysis

Descriptive statistics were summarised separately for participants with and without delirium within the dementia and no dementia groups, as well as for the full UK Biobank cohort that had available covariate and inpatient data for comparison. The two primary aims were to estimate (i) the impact of delirium on the risk of nursing home admission, and (ii) the extent to which nursing home admission occurred earlier in participants with delirium compared to those without, stratified by dementia group.

For aim (i), death was treated as a competing event for nursing home admission and modelled using Fine and Gray subdistribution hazard models [32]. Follow-up was measured in years from the index or control episode to nursing home admission, death or censoring (31 May 2022 for Wales; 31 August 2022 for Scotland; 31 October 2022 for England), whichever occurred first. Supplementary Tables 3 and 4 present the number of participants at risk, admitted to a nursing home, deceased or censored, stratified by delirium group. Models were adjusted for income and the Townsend Deprivation Index (standardised as a z-score), and results are presented as subdistribution hazard ratios (SHRs) with 95% confidence intervals.

For aim (ii), we used semiparametric accelerated failure time (AFT) models to estimate how much earlier delirium precipitates nursing home admission. AFT models [33] relate survival times across different exposure groups through time ratios, which quantify the extent to which an exposure accelerates (ratio < 1) or delays (ratio > 1) the time to an event. Unlike proportional hazards models, AFT models can be used to directly estimate differences in survival time, and the semiparametric approach improves robustness by relaxing distributional assumptions. The AFT models we adopted were adjusted for income and the Townsend Deprivation Index (standardised as a z-score) and fitted using a rank-based estimation [34, 35]. Results are expressed as the percentage hastened ([1 – time ratio] × 100%) in time to admission for the delirium group relative to the non-delirium group, with 95% confidence intervals. Absolute years hastened were then calculated by multiplying (1 – time ratio) by the time to nursing home admission in the no delirium group, derived from Kaplan–Meier curves at survival probabilities of 0.90 (3.75 years for no dementia) and 0.70 (3.13 years for dementia). Further details of these calculations are provided in the Supplementary Methods.

For both aims (i) and (ii), we then applied a landmark analysis to examine a potential dose–response relationship between the number of delirium episodes. Specifically, we assessed the number of delirium episodes occurring within the first 12 months of follow-up (Supplementary Table 5) in relation to nursing home admissions occurring after this 12-month period. The number of episodes was included as a categorical variable (0, 1 or ≥ 2) in the full sample, and as a continuous variable within the delirium cohorts only.

We tested the robustness of our findings with four sensitivity analyses. First, to minimise potential reverse causality, we excluded participants with less than 30 days of follow-up, as very early nursing home admission could reflect pre-existing functional decline rather than a consequence of delirium. Second, to address residual confounding from imperfect matching on hospital characteristics, we excluded the 10% of least well-matched pairs. Third, we excluded pairs in which delirium was coded as R41.0 to ensure the associations were not driven by non-specific confusion. Fourth, within the dementia subgroup, we excluded cases in which delirium and dementia were first recorded on the same day to minimise potential misclassification and reverse causality. Because sex is a plausible effect modifier, we also present estimates stratified by sex—even though Wald tests showed no interaction between delirium and sex, income or deprivation (all *P* > .40). Statistical significance was defined as a two-sided *P*-value <.05. Analyses were performed using R version 4.4.2.

## Results

Table 1 presents the characteristics of the two matched samples: the no dementia group (delirium *n* = 13 004; no delirium *n* = 13 004) and the dementia group (delirium *n* = 1790; no delirium *n* = 1790). Characteristics of the full sample with hospital inpatient data (*n* = 446 532) are included for comparison.

**Table 1:** Characteristics of the study sample.

		Participants without dementia		Participants with dementia	
Characteristics	Total eligible sample	No delirium group	Delirium group	No delirium group	Delirium group
Number of participants	445 982	13 004	13 004	1790	1790
Age (years), mean (SD)	60.3 (10.1)	71.4 (7.9)	71.6 (8.0)	75.6 (5.6)	75.7 (5.0)
Sex					
Men, *n* (%)	201 807 (45.3)	7225 (55.6)	7444 (57.2)	945 (52.8)	1014 (56.6)
Women, *n* (%)	244 175 (54.7)	5779 (44.5)	5560 (42.8)	845 (47.2)	776 (43.4)
Hospital frailty risk score, median (IQR)	0.4 (0, 1.1)	9.2 (6.2, 12.1)	11.7 (6.9, 18.5)	18.4 (13.6, 22.4)	20.4 (13.8, 29.1)
Low (<5), *n* (%)	373 650 (83.8)	2328 (17.9)	1841 (14.2)	141 (7.9)	24 (1.3)
Intermediate (5–15), *n* (%)	62 813 (14.1)	9184 (70.6)	6518 (50.1)	454 (25.4)	534 (29.8)
High (>15), *n* (%)	9519 (2.1)	1492 (11.5)	4645 (35.7)	1195 (66.8)	1232 (68.8)
Length of stay—index episode (days), median (IQR)	0.7 (0, 1.7)	4.0 (1.0, 11.0)	3.0 (1.0, 9.0)	3.0 (1.0, 9.0)	2.0 (1.0, 6.0)
Length of stay—intensive care unit					
None, *n* (%)	421 174 (94.4)	11 672 (89.7)	12 003 (92.3)	1766 (98.7)	1760 (98.3)
One day or longer, *n* (%)	24 808 (5.6)	1332 (10.2)	1001 (7.7)	24 (1.3)	30 (1.7)
Annual income, *n* (%)					
<£18 000	90 588 (20.3)	4157 (32.0)	4416 (34.0)	604 (33.7)	642 (35.9)
£18 000–30 999	97 940 (22.0)	3038 (23.4)	2923 (22.5)	409 (22.8)	397 (22.2)
£31 000–51 999	96 742 (21.7)	1943 (14.9)	1830 (14.1)	202 (11.3)	194 (10.8)
£52 000–100 000	72 390 (16.2)	1019 (7.8)	958 (7.4)	95 (5.3)	73 (4.1)
>£100 000	18 082 (4.1)	240 (1.8)	225 (1.7)	24 (1.3)	18 (1.0)
Do not know or prefer not to answer	70 240 (15.7)	2607 (20.0)	2652 (20.4)	456 (25.5)	466 (26.0)
Townsend Index of deprivation, median (IQR)	−2.1 (−3.6, 0.6)	−1.6 (−3.3, 1.6)	−1.6 (−3.3, 1.7)	−2.1 (−3.7, 1.0)	−1.6 (−3.4, 1.7)
Q1 (least deprived)	89 134 (20.0)	2085 (16.1)	2118 (16.3)	366 (20.4)	300 (16.8)
Q2	89 061 (20.0)	2348 (18.1)	2283 (17.6)	370 (20.7)	333 (18.6)
Q3	88 186 (19.8)	2448 (18.9)	2436 (18.8)	324 (18.1)	310 (17.3)
Q4	89 377 (20.0)	2651 (20.4)	2650 (20.4)	329 (18.4)	358 (20.0)
Q5 (most deprived)	90 224 (20.2)	3454 (26.6)	3503 (27.0)	401 (22.4)	487 (27.2)
Follow-up, person-years	–	37 953	38 070	2967	2487
Time to event, years, median (IQR)^a^	–	1.4 (0.3, 4.0)	1.3 (0.2, 3.9)	0.7 (0.1, 2.1)	0.6 (0.1, 2.1)
Time to event, range	–	0.01–26.6	0.01–26.4	0.01–21.9	0.01–20.0
Incident NH admission, *n* (%)^b^	–	1196 (9.2)	1504 (11.6)	494 (27.6)	610 (34.1)
Incidence rate of NH admission (per 100 person-years)	–	2.7	3.4	13.9	20.4
Died, *n* (%)	–	4239 (32.6)	5100 (39.2)	663 (37.0)	713 (39.8)

Abbreviations: SD, standard deviation; IQR, interquartile range.

a Time until nursing home admission, death or censoring.

b Cumulative incidence at 7 years.

### Delirium and nursing home admission in participants without dementia

**Figure 2:**
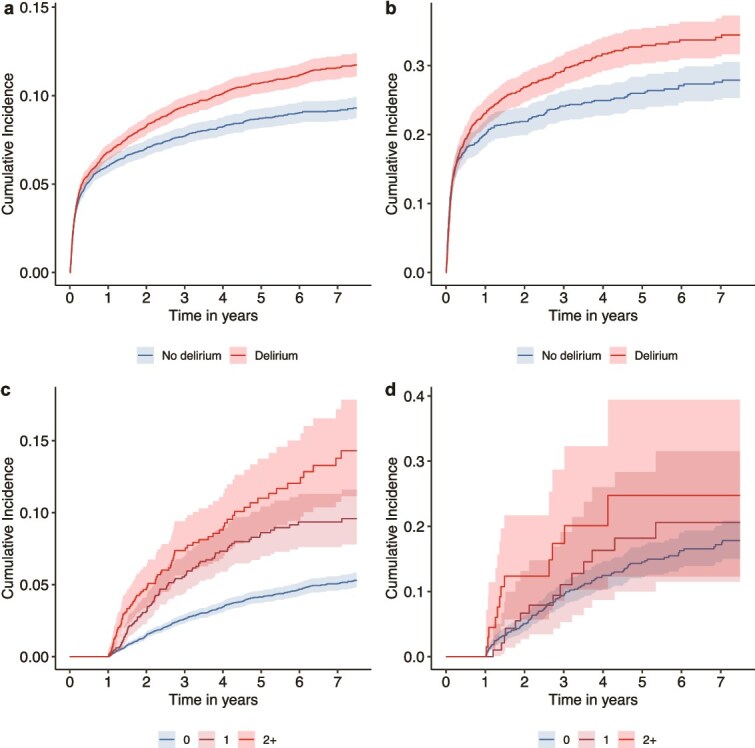
Cumulative incidence of nursing home admission in participants without dementia (panels A and C) and among those with dementia (panels B and D). Time axes were harmonised to 7 years. Panels C and D refer to the number of delirium episodes recorded during the 12-month landmark period. Shaded ribbons represent 95% confidence intervals. The cumulative incidence of the no-delirium (blue) lines differs between panels A and C (and between B and D) because panels C and D report findings from the 12-month landmark analysis, which includes only participants who remained free of nursing home admission and death during the first year of follow-up.

Over 7 years, the cumulative incidence of nursing home admission was higher in participants with delirium than in those without (11.6% vs. 9.2%; Figure 2a), corresponding to incidence rates of 3.4 and 2.7 per 100 person-years, respectively. Mortality over the same period was also higher in the delirium group (39.2% vs. 32.6%).

**Figure 3:**
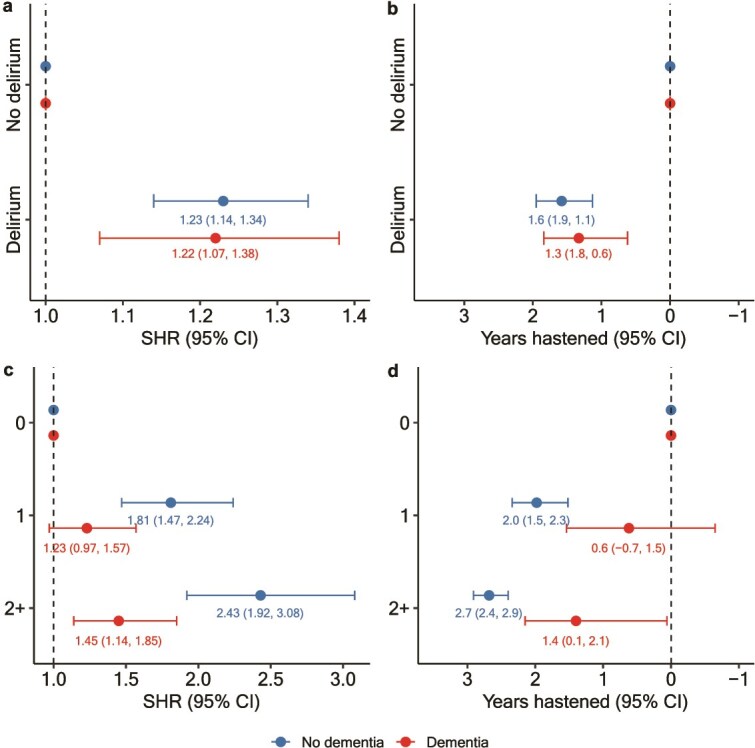
SHRs and years hastened for nursing home admission by delirium group (panels A and B). The delirium group is shown on the *y*-axis, and participants with and without dementia are highlighted in blue and red, respectively. SHRs and years hastened are also presented by delirium episode count within 12 months of the index or matched control episode (panels C and D). Years hastened were calculated by multiplying semiparametric accelerated failure time model time ratios for hastening by time to admission in the no-delirium group, derived from Kaplan–Meier curves at survival probabilities of 0.90 (no dementia) and 0.70 (dementia). All models adjust for income and Townsend deprivation index. Error bars indicate 95% confidence intervals.

Among participants without dementia, the incidence rate of nursing home admissions was 1.23 times higher in those with delirium compared to those without (SHR 1.23, 95% CI 1.14, 1.34), corresponding to a 23% increased risk of nursing home admission (Figure 3). In this group, nursing home admission occurred 42% (95% CI 30%, 52%) earlier, translating to a hastening of 1.6 years (95% CI 1.1, 1.9) at a survival probability of 0.90. The associations were stronger among men than women (Supplementary Table 6).

In the 12-month landmark analysis, increasing numbers of delirium episodes were associated with progressively higher risk and greater hastening of admission (Figure 3; Table 2), with similar findings among men and women (Supplementary Table 7). Among participants who experienced delirium at least once during the landmark period, each additional episode conferred a further 20% increase in risk (SHR 1.20, 95% CI 1.13, 1.27) and brought admission forward by 20% (95% CI 14%, 26%).

**Table 2:** Dose–response association between delirium episode count in the 12-month landmark period and time to first nursing home admission in participants with and without dementia.

Delirium episodes in 12-month landmark period	No dementia				Dementia			
	SHR (95% CI)^a^		Years hastened (95% CI)^b^		SHR (95% CI)^a^		Years hastened (95% CI)^c^	
0 (reference)								
1	1.81 (1.47, 2.24)	<0.001	2.0 (1.5, 2.3)	<0.001	1.23 (0.97, 1.57)	0.091	0.6 (−0.6, 1.5)	0.337
≥2	2.43 (1.92, 3.08)	<0.001	2.7 (2.4, 2.9)	<0.001	1.45 (1.14, 1.85)	0.002	1.4 (0.1, 2.2)	0.043

a Estimated using Fine and Gray competing risk models with death as competing event.

b Calculated as (1 – time ratio) × 3.75 years (the 3.75 years is the survival time for the no delirium no dementia group from Kaplan–Meier plots with survival probability of 0.90).

c Calculated as (1 – time ratio) × 3.13 years (the 3.13 years is the survival time for the no delirium dementia group from Kaplan–Meier plots with survival probability of 0.70).

### Delirium and nursing home admission in participants with dementia

Among participants with dementia, the 7-year cumulative incidence of nursing home admission was higher in those with delirium than in those without (34.1% vs. 27.6%; Figure 2b), corresponding to incidence rates of 20.4 and 13.9 per 100 person-years, respectively. Mortality was slightly higher in the delirium group (39.8% vs. 37.0%).

In participants with dementia, delirium was associated with a 22% higher risk of nursing home admission (SHR 1.22, 95% CI 1.07, 1.38; Figure 3). In this group, nursing home admission occurred 42% (95% CI 20%, 58%) earlier, translating to a hastening of 1.3 years (95% CI 0.6, 1.8) at a survival probability of 0.70. The associations were stronger among men than women (Supplementary Table 6).

In the 12-month landmark analysis, a greater number of delirium episodes was associated with progressively higher risk of subsequent nursing home admission and more pronounced hastening of admission (Figure 3 and Table 2). This dose–response relationship was not statistically significant in women (Supplementary Table 8). Among participants with delirium, additional episodes were not associated with risk of admission (SHR 1.09, 95% CI 0.88, 1.36) or time to admission (6%, 95% CI −11%, 20%).

### Sensitivity analysis

Excluding participants with short follow-up yielded similar estimates (Supplementary Tables 9–12). Excluding the 10% of least well-matched pairs and excluding non-specific delirium codes (R41.0) slightly strengthened the associations. Among participants with dementia, excluding same-day delirium episodes did not materially alter the results, which remained directionally consistent.

## Discussion

In this large, matched cohort drawn from the UK Biobank, a single episode of hospital-recorded delirium was associated with a one-fifth higher risk of nursing home admission in participants with and without dementia. Delirium was also associated with nursing home admission occurring ~40% earlier, bringing admission forward by more than a year, suggesting that delirium could represent a pivotal event in the trajectory of functional decline. These associations were dose-responsive, with each additional episode conferring further an increase in risk and shorter time to admission. Although multicomponent ward interventions can prevent hospital-acquired delirium [8], a substantial proportion of delirium has been observed to be present on admission [14]. Our findings, therefore, highlight delirium not only as a potentially modifiable factor whose prevention could delay—or even avert—nursing home admission, but also as a condition for which effective treatment strategies are urgently needed to mitigate downstream harm.

These findings extend and refine prior estimates of the association between delirium and nursing home admission [16–27, 29]. Our estimates lie toward the lower end of published reports, which have generally been based on smaller cohorts, shorter follow-up, and have not accounted for death as a competing risk. For example, a recent meta-analysis of 29 studies reported a pooled odds ratio of 2.80 for institutionalisation 1–24 months after delirium [10], while a meta-analysis focused on participants with dementia found a pooled relative risk of 1.53 at 12 months post-delirium [36]. By matching on key patient and hospital characteristics, adjusting for additional sociodemographic confounders and explicitly modelling death as a competing event, our study provides more conservative and robust estimates of the impact of delirium.

Earlier studies have reported that delirium confers a greater risk of nursing home admission in people with dementia than in those without [16, 19–21, 29]. In our cohort, which was slightly younger than many previously published dementia samples, delirium was associated with a similar relative elevation in risk regardless of pre-existing dementia. This suggests that the higher risk observed in dementia cohorts is largely attributable to dementia itself [5, 6] rather than to a delirium–dementia interaction. If so, these findings underscore the importance of preventing hospital-recorded delirium across older adult populations irrespective of pre-existing dementia. Although stratified analyses suggested somewhat stronger associations in men than in women, formal tests showed no statistical interaction by sex. The apparent differences are therefore more likely to reflect variation in statistical power or baseline subgroup characteristics than genuine effect modification.

Among participants without dementia, each delirium episode was associated with a further 20% higher risk and hastening of admission. Although a similar pattern was suggested among those with dementia, the associations were not statistically significant. This attenuation is consistent with recent evidence suggesting that the relative impact of delirium on adverse outcomes may be greater in individuals without pre-existing dementia or with better baseline cognition [14]. Nevertheless, alternative explanations include (i) diagnostic overshadowing, whereby new delirium episodes may be under-coded or overlooked once dementia is established; (ii) survivor bias, as individuals with the most severe delirium may not survive to experience subsequent episodes; and (iii) limited statistical power.

Several mechanisms could underlie these observations. Delirium occurs in the context of acute illness, which often precipitates or reveals a decline in functional status, giving rise to geriatric syndromes such as falls, immobility and functional decline [16, 19, 21, 36]. Even when cognitive symptoms of delirium resolve, a patient’s new degree of frailty may necessitate nursing home care [7, 10]. Acute delirium sets off a self-reinforcing cascade of damage through systemic and brain inflammation released from primed microglial and astrocyte cells, metabolic insufficiency characterised by hypoxaemia, impaired blood flow and glucose metabolism, and through vascular dysfunction from endothelial injury [7]. Together, these intertwined mechanisms could act as a functional ‘tipping point,’ so that each delirium episode hastens functional decline and brings forward the need for nursing home care in already vulnerable older adults. Delirium is also a recognised risk factor for incident dementia [11–13], with evidence of a dose–response association between recurrent episodes and subsequent dementia [11, 37]; dementia itself is a major driver of nursing home care [5, 6]. Together, the observed dose–response relationship suggests that repeated episodes of delirium might compound risk through both physical and cognitive deterioration.

Clinically, our results suggest that delirium is more than a transient hospital complication—it is a potentially modifiable driver of functional decline and earlier nursing home admission. Multicomponent interventions—including regular reorientation, early mobilisation, adequate hydration and nutrition and medication review—can be implemented by care teams and have been shown to prevent up to 40% of hospital-acquired delirium [15], and recent commentary highlights ongoing challenges in conducting delirium intervention trials in clinical practice [38]. Despite the high relevance of nursing home admission to patients, families and health systems—evidenced by the high personal [2–4] and societal costs of long-term care [1], including reduced quality of life and substantial healthcare expenditure—nursing home admission remains an underreported outcome in delirium epidemiology and intervention trials, which tend to focus on short term cognitive or functional outcomes.

### Strengths and limitations

This study used a large, population-based cohort and a granular, episode-level matched design accounting for key patient and hospital characteristics. By adjusting for sociodemographic confounders, restricting analyses to first delirium episodes, treating death as a competing risk and using accelerated failure-time models to estimate absolute differences in time to admission, we generated robust and clinically interpretable estimates. Diagnoses of delirium were identified using ICD-10 codes, which are known to underestimate true prevalence and incidence [39]. Diagnostic overlap with advanced dementia or Lewy body disease could also have contributed to underreporting [40]. To support temporal inference and reduce the risk of reverse causality from chronic or persistent delirium, we excluded participants with a history of delirium prior to their dementia diagnosis. We also excluded individuals with pre-existing dementia who were already living in nursing homes, which may have resulted in a healthier dementia subgroup and limited generalisability to more advanced dementia. While discharge destination data indicated admission to long-term nursing home units, we were unable to confirm the specific type or duration of care received. Nursing home admissions were identified from hospital discharge destination codes and therefore captured admissions only at discharge, potentially under-ascertaining admissions among individuals without subsequent hospitalisations. Prior care home residence was ascertainable only from earlier discharge records and may not have been identified in participants without previous hospital admissions. In the UK, nursing-home admission is usually permanent, whereas in the US, many nursing homes are short-stay skilled nursing facilities that receive patients once medically stabilised for rehabilitation. Our endpoint might therefore represent a more permanent transition than comparable US metrics, so effect sizes should be interpreted accordingly. While residual confounding cannot be excluded, sensitivity analyses excluding the least well-matched pairs produced slightly stronger associations, supporting the robustness of our findings. Frailty was assessed using the hospital frailty risk score; the frailty index [41] in the UK Biobank had been collected a mean of 9 years before the participants’ index admissions, limiting its suitability for this study. Our cohort comprised UK Biobank participants with at least one hospital admission and is not representative of the general population [42]. The dementia subsample was slightly younger than many previously published dementia cohorts; therefore, our findings most directly apply to hospitalised, community-dwelling older adults with dementia and may be less generalisable to older individuals in long-term care or those with more advanced disease.

## Conclusions

Hospital-recorded delirium was consistently associated with both whether and when older adults were admitted to nursing homes. It was associated with a roughly one-fifth higher risk of admission in people with and without dementia and, on average, precipitated admission more than a year earlier. The dose–response gradient observed in those without dementia suggests that each additional episode compounds risk, underscoring the cumulative toll of delirium on functional reserve. Systematic delirium prevention could represent a tangible opportunity to delay—or even avert—transitions to long term care and improve quality of life for older adults.

## Supplementary Material

aa-25-3617-File002_afag151

## Research Data Transparency and Availability

UK Biobank is an open access resource. Bona fide researchers can apply to use the UK Biobank dataset by registering and applying at http://ukbiobank.ac.uk/register-apply/.
