# Supplementary material for: Delirium and incident nursing home admission among people with and without dementia
Source: Age Ageing. 2026 May 30;55(5):afag151. doi: 10.1093/ageing/afag151 (PMC13221970; doi:10.1093/ageing/afag151)
Supplement: aa-25-3617-File002_afag151 [file aa-25-3617-file002_afag151.docx]

Delirium and incident nursing home admission among people with and without dementia

Supplementary Data

Supplementary Methods Estimation of time hastened

Supplementary Table 1. List of ICD-10 codes used to identify dementia in the UK Biobank cohort.

Supplementary Table 2. List of ICD-10 codes used to identify delirium in the UK Biobank cohort.

Supplementary Table 3. Numbers at risk, with primary event, with competing event, and numbers censored in the no delirium and delirium groups in participants without dementia.

Supplementary Table 4. Numbers at risk, with primary event, with competing event, and numbers censored in the no delirium and delirium groups in participants with dementia.

Supplementary Table 5. Distribution of delirium episodes during the 12-month landmark period in participants with and without dementia.

Supplementary Table 6. Association of delirium with the risk and timing of nursing-home admission in participants with and without dementia, stratified by sex.

Supplementary Table 7. Dose-response association between the number of delirium episodes during the 12-month landmark period and subsequent nursing-home admission in participants without dementia, stratified by sex.

Supplementary Table 8. Dose-response association between the number of delirium episodes during the 12-month landmark period and subsequent nursing-home admission in participants with dementia, stratified by sex.

Supplementary Table 9. Association of delirium with the risk and timing of nursing-home admission in participants without dementia: primary and sensitivity analyses.

Supplementary Table 10. Dose-response relationship between the number of delirium episodes during the 12-month landmark period and subsequent nursing-home admission in participants without dementia: primary and sensitivity analyses.

Supplementary Table 11. Association of delirium with the risk and timing of nursing-home admission in participants with dementia: primary and sensitivity analyses.

Supplementary Table 12. Dose-response relationship between the number of delirium episodes during the 12-month landmark period and subsequent nursing-home admission in participants with dementia: primary and sensitivity analyses.

Supplementary Methods. Estimation of time hastened.

In this paper, estimation of the time hastened attributable to delirium proceeds in three steps: (i) estimation of the delirium effect in a semiparametric accelerated failure time (AFT) model using a rank-based method, (ii) estimation of reference survival curves using Kaplan–Meier (KM) methods, and (iii) calculation of time hastened at selected survival probabilities.

Step 1. Estimation of the delirium coefficient(s) using a rank-based AFT model

We estimate the delirium effect (delirium versus non-delirium) separately within dementia strata, yielding one coefficient for participants without dementia, $\hat{\beta}_{1}$, and one coefficient for participants with dementia, $\hat{\beta}_{2}$. These coefficients are obtained using a rank-based estimator for the semiparametric AFT model (details provided in main text references^34,35^).

The AFT model implies a multiplicative effect of delirium on survival time (a proportional survival-time assumption) within each dementia stratum. Specifically, within the no-dementia stratum the time ratio for delirium versus non-delirium is assumed constant and determined by $\hat{\beta}_{1}$, and within the dementia stratum the corresponding constant time ratio is determined by $\hat{\beta}_{2}$.

Step 2. Estimation of reference survival curves using Kaplan–Meier

We estimate the survival function for the reference (non-delirium) group separately within each dementia stratum using the KM estimator:

$\hat{S}_{1}(t)$: non-delirium, no-dementia subgroup;

$\hat{S}_{2}(t)$: non-delirium, dementia subgroup.

The KM estimator is a nonparametric estimate of the survival curve and is piecewise constant between event times. For a given survival probability $u\in[0,1]$, the corresponding estimated survival time (quantile) is obtained by inversion:

$\hat{t}=\hat{S}^{-1}\left( u \right)$.

In this paper, we report:

$\hat{t}_{1}=\hat{S}_{1}^{-1}\left( u_{1} \right)$, $u_{1}=0.90$, $\hat{t}_{1}=3.75$years,

$\hat{t}_{2}=\hat{S}_{2}^{-1}\left( u_{2} \right)$, $u_{2}=0.70$, $\hat{t}_{2}=3.13$years.

The choices $u_{1}=0.90$ and $u_{2}=0.70$ were driven by data availability (adequate support in the observed follow-up for stable estimation in each subgroup). We focus on pointwise interpretations at these survival probabilities rather than attempting to interpret time hastened over the full range of $u$, because the absolute time difference implied by an AFT model depends on the underlying survival time (and therefore varies with $u$).

Step 3. Calculation of percentage hastened and time hastened

Under the AFT model parameterisation used here, the time ratio comparing delirium to non-delirium (holding other covariates fixed, including dementia stratum, income, and Townsend Deprivation Index) is $\exp\left( -\beta\right)$.

Hence, the corresponding percentage hastened (does not require step 2) is

$[1-exp \left( -\beta\right)]\times100\%$,

and the time hastened at a reference survival time t is

$[1-exp \left( -\beta\right)]t$.

Illustration (no-dementia subgroup). The percentage hastened due to delirium is

$[1-exp \left( -\hat{\beta}_{1} \right)]\times100\%$,

and the years hastened due to delirium at survival probability $u_{1}=0.90$ is

$[1-exp \left( -\hat{\beta}_{1} \right)]\cdot\hat{S}_{1}^{-1}\left( u_{1} \right)$.

(Analogous expressions apply to the dementia subgroup by replacing $\hat{\beta}_{1}$ and $\hat{S}_{1}$ with $\hat{\beta}_{2}$ and $\hat{S}_{2}$.)

References:

34. Chiou S, Kang S, Yan J. Rank-based estimating equations with general weight for accelerated failure time models: an induced smoothing approach. *Stat Med*. 2015;34(9):1495-1510. doi:10.1002/SIM.6415

35. Chiou SH, Kang S. Fitting Accelerated Failure Time Models in Routine Survival Analysis with R Package aftgee. *J Stat Softw*. 2014;61(11):1-23. doi:10.18637/JSS.V061.I11

Supplementary Table 1. List of ICD-10 codes used to identify dementia in the UK Biobank cohort.

| **ICD-10 code** | **Description** |
| --- | --- |
|  |  |
| F00.1 | Dementia in Alzheimer’s disease with late onset |
| F00.2 | Dementia in Alzheimer’s disease, atypical or mixed type |
| F00.9 | Dementia in Alzheimer’s disease, unspecified |
| G30.1 | Alzheimer’s disease with late onset |
| G30.8 | Other Alzheimer’s disease |
| G30.9 | Alzheimer’s disease, unspecified |
| F01.0 | Vascular dementia of acute onset |
| F01.1 | Multi-infarct dementia |
| F01.2 | Subcortical vascular dementia |
| F01.3 | Mixed cortical and subcortical vascular dementia |
| F01.8 | Other vascular dementia |
| F01.9 | Vascular dementia, unspecified |
| I67.3 | Binswanger disease |
| F02.0 | Dementia in Pick disease |
| F02.3 | Dementia in Parkinson’s disease |
| F03 | Unspecified dementia |
| F05.1 | Delirium superimposed on dementia |
| G31.0 | Circumscribed brain atrophy |
| G31.1 | Senile degeneration of the brain, not elsewhere classified |
| G31.8 | Other specified degenerative diseases of the nervous system |
|  |  |

Supplementary Table 2. List of ICD-10 codes used to define delirium in the UK Biobank cohort.

| **ICD-10 code** | **Description** |
| --- | --- |
|  |  |
| F05.0 | Delirium not superimposed on dementia, so described |
| F05.8 | Other delirium |
| F05.9 | Delirium, unspecified |
| R41.0 | Disorientation, unspecified ‘Confusion NOS’ |
|  |  |

Supplementary Table 3. Numbers at risk, with primary event, with competing event, and numbers censored in the no delirium and delirium groups in participants without dementia.

| No delirium group | | | | |  | Delirium group | | | | |
| --- | --- | --- | --- | --- | --- | --- | --- | --- | --- | --- |
| Time (years) | At risk | Primary event | Competing event | Censored |  | Time (years) | At risk | Primary event | Competing event | Censored |
| 0 | 13004 | 544 | 2307 | 529 |  | 0 | 13004 | 716 | 3132 | 450 |
| 1 | 9624 | 638 | 2848 | 1978 |  | 1 | 8706 | 938 | 3972 | 1820 |
| 2 | 7540 | 678 | 3083 | 3170 |  | 2 | 6274 | 1052 | 4336 | 2807 |
| 3 | 6073 | 695 | 3230 | 4092 |  | 3 | 4809 | 1122 | 4584 | 3561 |
| 4 | 4987 | 710 | 3320 | 4935 |  | 4 | 3737 | 1171 | 4742 | 4170 |
| 5 | 4039 | 722 | 3389 | 5547 |  | 5 | 2921 | 1194 | 4859 | 4675 |
| 6 | 3346 | 734 | 3428 | 5968 |  | 6 | 2276 | 1217 | 4937 | 4996 |
| 7 | 2874 | 738 | 3457 | 6340 |  | 7 | 1854 | 1228 | 4985 | 5261 |
| 8 | 2469 | 740 | 3485 | 6660 |  | 8 | 1530 | 1237 | 5038 | 5467 |
| 9 | 2119 | 745 | 3511 | 6964 |  | 9 | 1262 | 1250 | 5067 | 5669 |
| 10 | 1784 | 746 | 3537 | 7219 |  | 10 | 1018 | 1255 | 5089 | 5805 |
| 11 | 1502 | 747 | 3553 | 7407 |  | 11 | 855 | 1263 | 5112 | 5924 |
| 12 | 1297 | 751 | 3563 | 7562 |  | 12 | 705 | 1265 | 5124 | 6040 |
| 13 | 1128 | 753 | 3571 | 7729 |  | 13 | 575 | 1267 | 5136 | 6129 |
| 14 | 951 | 754 | 3578 | 7856 |  | 14 | 472 | 1270 | 5145 | 6201 |
| 15 | 816 | 755 | 3587 | 7983 |  | 15 | 388 | 1272 | 5150 | 6260 |
| 16 | 679 | 756 | 3591 | 8128 |  | 16 | 322 | 1274 | 5153 | 6310 |
| 17 | 529 | 756 | 3592 | 8229 |  | 17 | 267 | 1274 | 5158 | 6356 |
| 18 | 429 | 756 | 3597 | 8306 |  | 18 | 216 | 1276 | 5163 | 6391 |
| 19 | 345 | 756 | 3598 | 8370 |  | 19 | 174 | 1277 | 5167 | 6420 |
| 20 | 280 | 756 | 3601 | 8433 |  | 20 | 140 | 1277 | 5169 | 6447 |
| 21 | 214 | 756 | 3604 | 8479 |  | 21 | 111 | 1277 | 5172 | 6472 |
| 22 | 165 | 756 | 3606 | 8528 |  | 22 | 83 | 1277 | 5174 | 6498 |
| 23 | 114 | 756 | 3607 | 8571 |  | 23 | 55 | 1277 | 5175 | 6516 |
| 24 | 70 | 756 | 3607 | 8607 |  | 24 | 36 | 1277 | 5176 | 6534 |
| 25 | 34 | 756 | 3607 | 8632 |  | 25 | 17 | 1277 | 5177 | 6545 |
| 26 | 9 | 756 | 3607 | 8641 |  | 26 | 5 | 1277 | 5177 | 6549 |
| 27 | 0 |  |  |  |  | 27 | 1 | 1277 | 5177 | 6550 |
|  |  |  |  |  |  |  |  |  |  |  |

Supplementary Table 4. Numbers at risk, with primary event, with competing event, and numbers censored in the no delirium and delirium groups in participants with dementia.

| No delirium group | | | | |  | Delirium group | | | | |
| --- | --- | --- | --- | --- | --- | --- | --- | --- | --- | --- |
| Time (years) | At risk | Primary event | Competing event | Censored |  | Time (years) | At risk | Primary event | Competing event | Censored |
| 0 | 1790 | 333 | 387 | 103 |  | 0 | 1790 | 340 | 376 | 70 |
| 1 | 967 | 382 | 514 | 317 |  | 1 | 1004 | 432 | 524 | 221 |
| 2 | 577 | 400 | 579 | 464 |  | 2 | 613 | 463 | 602 | 343 |
| 3 | 347 | 410 | 619 | 558 |  | 3 | 382 | 485 | 659 | 430 |
| 4 | 203 | 414 | 637 | 609 |  | 4 | 216 | 497 | 695 | 480 |
| 5 | 130 | 418 | 647 | 646 |  | 5 | 118 | 502 | 712 | 510 |
| 6 | 79 | 422 | 657 | 666 |  | 6 | 66 | 504 | 721 | 528 |
| 7 | 45 | 423 | 663 | 672 |  | 7 | 37 | 506 | 727 | 536 |
| 8 | 32 | 423 | 667 | 677 |  | 8 | 21 | 507 | 729 | 542 |
| 9 | 23 | 423 | 669 | 684 |  | 9 | 12 | 507 | 730 | 544 |
| 10 | 14 | 423 | 671 | 687 |  | 10 | 9 | 507 | 731 | 546 |
| 11 | 9 | 423 | 671 | 689 |  | 11 | 6 | 507 | 731 | 547 |
| 12 | 7 | 423 | 671 | 692 |  | 12 | 5 | 507 | 731 | 550 |
| 13 | 4 | 423 | 671 | 693 |  | 13 | 2 | 507 | 731 | 551 |
| 14 | 3 | 423 | 671 | 696 |  | 14 | 1 | 507 | 731 | 552 |
|  |  |  |  |  |  |  |  |  |  |  |

Supplementary Table 5. Distribution of delirium episodes during the 12-month landmark period in participants with and without dementia.

|  |  | Delirium episodes in 12-month landmark period | | | | |
| --- | --- | --- | --- | --- | --- | --- |
|  |  | 0 |  | 1 |  | 2 or more |
|  |  |  |  |  |  |  |
| Participants without dementia (n=7306)^a^ |  | 4,801 (65.7) |  | 1,538 (21.1) |  | 967 (13.2) |
| Participants with dementia (n=747)^b^ |  | 576 (77.1) |  | 104 (13.9) |  | 67 (9.0) |
|  |  |  |  |  |  |  |

^a^ A total of 5,698 participants were excluded from the landmark analysis because they had been admitted to a nursing home, had died, or had been censored during the first 12 months of follow-up.

^b^ A total of 1,043 participants were excluded from the landmark analysis because they had been admitted to a nursing home, had died, or had been censored during the first 12 months of follow-up.

Supplementary Table 6. Association of delirium with the risk and timing of nursing-home admission in participants with and without dementia, stratified by sex.

|  |  | Participants without dementia (delirium group vs. no delirium) | | | | | | | | |
| --- | --- | --- | --- | --- | --- | --- | --- | --- | --- | --- |
|  |  |  | Delirium group | |  | Delirium group | |  | Delirium group | |
|  |  |  | SHR (95% CI)^a^ | |  | % hastened (95% CI)^b^ | |  | Years hastened (95% CI)^c^ | |
| Sex |  |  |  |  |  |  |  |  |  |  |
| Men |  |  | 1.33 (1.18, 1.48) | <0.001 |  | 52 (37, 63) | <0.001 |  | 1.9 (1.4, 2.4) | <0.001 |
| Women |  |  | 1.14 (1.01, 1.28) | 0.037 |  | 29 (8, 46) | 0.009 |  | 1.1 (0.3, 1.7) | 0.009 |
|  |  |  |  |  |  |  |  |  |  |  |
|  |  | Participants with dementia (delirium group vs. no delirium) | | | | | | | | |
|  |  |  | Delirium group | |  | Delirium group | |  | Delirium group | |
|  |  |  | SHR (95% CI)^a^ | |  | % hastened (95% CI)^b^ | |  | Years hastened (95% CI)^d^ | |
| Sex |  |  |  |  |  |  |  |  |  |  |
| Men |  |  | 1.25 (1.04, 1.49) | 0.017 |  | 43 (13, 63) | 0.010 |  | 1.3 (0.4, 2.0) | 0.010 |
| Women |  |  | 1.19 (0.99, 1.44) | 0.070 |  | 41 (0, 65) | 0.053 |  | 1.3 (0, 2.0) | 0.053 |
|  |  |  |  |  |  |  |  |  |  |  |

^a^ Estimated using Fine and Gray competing risk models with death as competing event.

^b^ Estimated using rank-based accelerated failure time models. Coefficients are [(1 – time ratios) × 100%], where time ratios are exp(β) coefficients.

^c^ Calculated as (1 – time ratio) × 3.75 years (the 3.75 years is the survival time for the no delirium no dementia group from Kaplan-Meier plots with survival probability of 0.90).

^d^ Calculated as (1 – time ratio) × 3.13 years (the 3.13 years is the survival time for the no delirium dementia group from Kaplan-Meier plots with survival probability of 0.70).

Supplementary Table 7. Dose-response association between the number of delirium episodes during the 12-month landmark period and subsequent nursing-home admission in participants without dementia, stratified by sex.

|  |  | Participants without dementia | | | | | | |
| --- | --- | --- | --- | --- | --- | --- | --- | --- |
|  |  |  |  | Delirium episodes in 12-month landmark period | | | |  |
|  |  | 0 |  | 1 | |  | 2 or more | |
|  |  |  |  |  |  |  |  |  |
|  |  |  |  | SHR (95% CI)^a^ | p |  | SHR (95% CI)^a^ | p |
| Sex |  |  |  |  |  |  |  |  |
| Men |  | Ref. |  | 1.72 (1.27, 2.33) | <0.001 |  | 2.64 (1.92, 3.64) | <0.001 |
| Women |  | Ref. |  | 1.94 (1.44, 2.61) | <0.001 |  | 2.24 (1.57, 3.19) | <0.001 |
|  |  |  |  |  |  |  |  |  |
|  |  |  |  | % hastened (95% CI)^b^ | p |  | % hastened (95% CI)^b^ | p |
| Sex |  |  |  |  |  |  |  |  |
| Men |  | Ref. |  | 52 (32, 67) | <0.001 |  | 76 (66, 83) | <0.001 |
| Women |  | Ref. |  | 53 (34, 66) | <0.001 |  | 66 (52, 76) | <0.001 |
|  |  |  |  |  |  |  |  |  |
|  |  |  |  | Years hastened (95% CI)^c^ | p |  | Years hastened (95% CI)^c^ | p |
| Sex |  |  |  |  |  |  |  |  |
| Men |  | Ref. |  | 2.0 (1.2, 2.5) | <0.001 |  | 2.9 (2.5, 3.1) | <0.001 |
| Women |  | Ref. |  | 2.0 (1.3, 2.5) | <0.001 |  | 2.5 (2.0, 2.9) | <0.001 |
|  |  |  |  |  |  |  |  |  |

^a^ Estimated using Fine and Gray competing risk models with death as competing event.

^b^ Estimated using rank-based accelerated failure time models. Coefficients are [(1 – time ratios) × 100%], where time ratios are exp(β) coefficients.

^c^ Calculated as (1 – time ratio) × 3.75 years (the 3.75 years is the survival time for the no delirium no dementia group from Kaplan-Meier plots with survival probability of 0.90).

Supplementary Table 8. Dose-response association between the number of delirium episodes during the 12-month landmark period and subsequent nursing-home admission in participants with dementia, stratified by sex.

|  |  | Participants with dementia | | | | | | |
| --- | --- | --- | --- | --- | --- | --- | --- | --- |
|  |  |  |  | Delirium episodes in 12-month landmark period | | | |  |
|  |  | 0 |  | 1 | |  | 2 or more | |
|  |  |  |  |  |  |  |  |  |
|  |  |  |  | SHR (95% CI)^a^ | p |  | SHR (95% CI)^a^ | p |
| Sex |  |  |  |  |  |  |  |  |
| Men |  | Ref. |  | 1.04 (0.72, 1.49) | 0.85 |  | 1.48 (1.10, 1.99) | 0.010 |
| Women |  | Ref. |  | 1.46 (1.06, 2.02) | 0.021 |  | 1.47 (0.97, 2.23) | 0.068 |
|  |  |  |  |  |  |  |  |  |
|  |  |  |  | % hastened (95% CI)^b^ | p |  | % hastened (95% CI)^b^ | p |
| Sex |  |  |  |  |  |  |  |  |
| Men |  | Ref. |  | 0.0 (-0.8, 0.5) | 0.99 |  | 50 (2, 74) | 0.043 |
| Women |  | Ref. |  | 45 (-20, 75) | 0.132 |  | 10 (-169, 70) | 0.852 |
|  |  |  |  |  |  |  |  |  |
|  |  |  |  | Years hastened (95% CI)^c^ | p |  | Years hastened (95% CI)^c^ | p |
| Sex |  |  |  |  |  |  |  |  |
| Men |  | Ref. |  | 0.0 (-2.5, 1.6) | 0.99 |  | 1.6 (0.6, 2.3) | 0.043 |
| Women |  | Ref. |  | 1.4 (-0.6, 2.4) | 0.132 |  | 0.3 (-5.3, 2.2) | 0.852 |
|  |  |  |  |  |  |  |  |  |

^a^ Estimated using Fine and Gray competing risk models with death as competing event.

^b^ Estimated using rank-based accelerated failure time models. Coefficients are [(1 – time ratios) × 100%], where time ratios are exp(β) coefficients.

^c^ Calculated as (1 – time ratio) × 3.13 years (the 3.13 years is the survival time for the no delirium dementia group from Kaplan-Meier plots with survival probability of 0.70).

Supplementary Table 9. Association of delirium with the risk and timing of nursing-home admission in participants without dementia: primary and sensitivity analyses.

|  |  | Participants without dementia (delirium group vs. no delirium) | | | | | | | | |
| --- | --- | --- | --- | --- | --- | --- | --- | --- | --- | --- |
|  |  |  | Delirium group | |  | Delirium group | |  | Delirium group | |
|  |  |  | SHR (95% CI)^a^ | |  | % hastened (95% CI)^b^ | |  | Years hastened (95% CI)^c^ | |
|  |  |  |  |  |  |  |  |  |  |  |
| Primary analysis |  |  | 1.23 (1.14, 1.34) | <0.001 |  | 42 (30, 52) | <0.001 |  | 1.6 (1.1, 1.9) | <0.001 |
| Sensitivity analysis one^d^ |  |  | 1.24 (1.14, 1.35) | <0.001 |  | 48 (35, 57) | <0.001 |  | 1.8 (1.3, 2.1) | <0.001 |
| Sensitivity analysis two^e^ |  |  | 1.36 (1.24, 1.49) | <0.001 |  | 51 (40, 60) | <0.001 |  | 1.9 (1.5, 2.3) | <0.001 |
| Sensitivity analysis three^f^ |  |  | 1.33 (1.21, 1.45) | <0.001 |  | 54 (45, 63) | <0.001 |  | 2.0 (1.7, 2.3) | <0.001 |
|  |  |  |  |  |  |  |  |  |  |  |

^a^ Estimated using Fine and Gray competing risk models with death as competing event.

^b^ Estimated using rank-based accelerated failure time models. Coefficients are [(1 – time ratios) × 100%], where time ratios are exp(β) coefficients.

^c^ Calculated as (1 – time ratio) × 3.75 years (the 3.75 years is the survival time for the no delirium no dementia group from Kaplan-Meier plots with survival probability of 0.90).

^d^ Excluding participants who died or were admitted to a nursing home on the same episode as index or control episode.

^e^ Excluding the 10% least well-matched pairs based on Eucledian distance values.

^f^ Excluding pairs where the delirium case had an ICD-10 diagnosis of R41.0.

Supplementary Table 10. Dose-response relationship between the number of delirium episodes during the 12-month landmark period and subsequent nursing-home admission in participants without dementia: primary and sensitivity analyses.

|  |  | Participants without dementia | | | | | | |
| --- | --- | --- | --- | --- | --- | --- | --- | --- |
|  |  |  |  | Delirium episodes in 12-month landmark period | | | |  |
|  |  | 0 |  | 1 | |  | 2 or more | |
|  |  |  |  |  |  |  |  |  |
|  |  |  |  | SHR (95% CI)^a^ | p |  | SHR (95% CI)^a^ | p |
| Primary analysis |  | Ref. |  | 1.81 (1.47, 2.24) | <0.001 |  | 2.43 (1.92, 3.08) | <0.001 |
| Sensitivity analysis one^d^ |  | Ref. |  | 1.82 (1.47, 2.25) | <0.001 |  | 2.44 (1.92, 2.25) | <0.001 |
| Sensitivity analysis two^e^ |  | Ref. |  | 1.95 (1.55, 2.44) | <0.001 |  | 2.46 (1.89, 3.21) | <0.001 |
| Sensitivity analysis three^f^ |  | Ref |  | 2.02 (1.62, 2.53) | <0.001 |  | 2.43 (1.88, 3.14) | <0.001 |
|  |  |  |  |  |  |  |  |  |
|  |  |  |  | % hastened (95% CI)^b^ | p |  | % hastened (95% CI)^b^ | p |
| Primary analysis |  | Ref. |  | 53 (41, 62) | <0.001 |  | 72 (64, 78) | <0.001 |
| Sensitivity analysis one^d^ |  | Ref. |  | 53 (41, 62) | <0.001 |  | 72 (63, 78) | <0.001 |
| Sensitivity analysis two^e^ |  | Ref. |  | 55 (42, 65) | <0.001 |  | 72 (64, 79) | <0.001 |
| Sensitivity analysis three^f^ |  | Ref |  | 56 (44, 66) | <0.001 |  | 70 ( |  |
|  |  |  |  |  |  |  |  |  |
|  |  |  |  | Years hastened (95% CI)^c^ | p |  | Years hastened (95% CI)^c^ | p |
| Primary analysis |  | Ref. |  | 2.0 (1.5, 2.3) | <0.001 |  | 2.7 (2.4, 2.9) | <0.001 |
| Sensitivity analysis one^d^ |  | Ref. |  | 2.0 (1.5, 2.3) | <0.001 |  | 2.7 (2.4, 2.9) | <0.001 |
| Sensitivity analysis two^e^ |  | Ref. |  | 2.1 (1.6, 2.4) | <0.001 |  | 2.7 (2.4, 3.0) | <0.001 |
| Sensitivity analysis three^f^ |  | Ref |  | 2.1 (1.6, 2.5) | <0.001 |  | 2.6 (2.3, 2.9) | <0.001 |
|  |  |  |  |  |  |  |  |  |

^a^ Estimated using Fine and Gray competing risk models with death as competing event.

^b^ Estimated using rank-based accelerated failure time models. Coefficients are [(1 – time ratios) × 100%], where time ratios are exp(β) coefficients.

^c^ Calculated as (1 – time ratio) × 3.75 years (the 3.75 years is the survival time for the no delirium no dementia group from Kaplan-Meier plots with survival probability of 0.90).

^d^ Excluding participants who died or were admitted to a nursing home on the same episode as index or control episode.

^e^ Excluding the 10% least well-matched pairs based on Eucledian distance values.

^f^ Excluding pairs where the delirium case had an ICD-10 diagnosis of R41.0.

Supplementary Table 11. Association of delirium with the risk and timing of nursing-home admission in participants with dementia: primary and sensitivity analyses.

|  |  | Participants with dementia (delirium group vs. no delirium) | | | | | | | | |
| --- | --- | --- | --- | --- | --- | --- | --- | --- | --- | --- |
|  |  |  | Delirium group | |  | Delirium group | |  | Delirium group | |
|  |  |  | SHR (95% CI)^a^ | |  | % hastened (95% CI)^b^ | |  | Years hastened (95% CI)^c^ | |
|  |  |  |  |  |  |  |  |  |  |  |
| Primary analysis |  |  | 1.22 (1.07, 1.38) | 0.003 |  | 42 (20, 58) | 0.001 |  | 1.3 (0.6, 1.8) | 0.001 |
| Sensitivity analysis one^d^ |  |  | 1.23 (1.08, 1.41) | 0.002 |  | 45 (23, 60) | <0.001 |  | 1.4 (0.7, 1.9) | <0.001 |
| Sensitivity analysis two^e^ |  |  | 1.26 (1.10, 1.45) | 0.001 |  | 48 (25, 64) | <0.001 |  | 1.5 (0.8, 2.0) | <0.001 |
| Sensitivity analysis three^f^ |  |  | 1.20 (1.04, 1.38) | 0.013 |  | 51 (22, 69) | 0.003 |  | 1.6 (0.7, 2.2) | 0.003 |
| Sensitivity analysis four^g^ |  |  | 1.19 (1.03, 1.39) | 0.022 |  | 47 (20, 65) | 0.003 |  | 1.5 (0.6, 2.0) | 0.003 |
|  |  |  |  |  |  |  |  |  |  |  |

^a^ Estimated using Fine and Gray competing risk models with death as competing event.

^b^ Estimated using rank-based accelerated failure time models. Coefficients are [(1 – time ratios) × 100%], where time ratios are exp(β) coefficients.

^c^ Calculated as (1 – time ratio) × 3.13 years (the 3.13 years is the survival time for the no delirium dementia group from Kaplan-Meier plots with survival probability of 0.70).

^d^ Excluding participants who died or were admitted to a nursing home on the same episode as index or control episode.

^e^ Excluding the 10% least well-matched pairs based on Eucledian distance values.

^f^ Excluding pairs where the delirium case had an ICD-10 diagnosis of R41.0.

^g^ Excluding participants who had their first diagnosis of delirium on the same day as their first diagnosis of dementia.

Supplementary Table 12. Dose-response relationship between the number of delirium episodes during the 12-month landmark period and subsequent nursing-home admission in participants with dementia: primary and sensitivity analyses.

|  |  | Participants with dementia | | | | | | |
| --- | --- | --- | --- | --- | --- | --- | --- | --- |
|  |  |  |  | Delirium episodes in 12-month landmark period | | | |  |
|  |  | 0 |  | 1 | |  | 2 or more | |
|  |  |  |  |  |  |  |  |  |
|  |  |  |  | SHR (95% CI)^a^ | p |  | SHR (95% CI)^a^ | p |
| Primary analysis |  | Ref. |  | 1.23 (0.97, 1.57) | 0.091 |  | 1.45 (1.14, 1.85) | 0.002 |
| Sensitivity analysis one^d^ |  | Ref. |  | 1.22 (0.95, 1.55) | 0.12 |  | 1.46 (1.14, 1.55) | 0.002 |
| Sensitivity analysis two^e^ |  | Ref. |  | 1.22 (0.95, 1.58) | 0.13 |  | 1.42 (1.10, 1.83) | 0.008 |
| Sensitivity analysis three^f^ |  | Ref. |  | 0.82 (0.38, 1.74) | 0.60 |  | 1.92 (1.01, 3.63) | 0.045 |
| Sensitivity analysis four^g^ |  | Ref. |  | 1.20 (0.86, 1.67) | 0.29 |  | 1.69 (1.25, 2.27) | <0.001 |
|  |  |  |  |  |  |  |  |  |
|  |  |  |  | % hastened (95% CI)^b^ | p |  | % hastened (95% CI)^b^ | p |
| Primary analysis |  | Ref. |  | 20 (-21, 49) | 0.337 |  | 42 (2, 69) | 0.043 |
| Sensitivity analysis one^d^ |  | Ref. |  | 20 (-28, 50) | 0.351 |  | 45 (-2, 70) | 0.056 |
| Sensitivity analysis two^e^ |  | Ref. |  | 10 (-54, 47) | 0.71 |  | 47 (-8, 74) | 0.078 |
| Sensitivity analysis three^f^ |  | Ref. |  | -55 (-261, 33) | 0.307 |  | 63 (1, 85) | 0.036 |
| Sensitivity analysis four^g^ |  | Ref. |  | 0 (-114, 53) | 0.99 |  | 50 (-22, 80) | 0.128 |
|  |  |  |  |  |  |  |  |  |
|  |  |  |  | Years hastened (95% CI)^c^ | p |  | Years hastened (95% CI)^c^ | p |
| Primary analysis |  | Ref. |  | 0.6 (-0.7, 1.5) | 0.337 |  | 1.3 (0.1, 2.2) | 0.043 |
| Sensitivity analysis one^d^ |  | Ref. |  | 0.6 (-0.9, 1.6) | 0.351 |  | 1.4 (-0.1, 2.2) | 0.056 |
| Sensitivity analysis two^e^ |  | Ref. |  | 0.3 (-1.7, 1.5) | 0.71 |  | 1.5 (-0.3, 2.3) | 0.078 |
| Sensitivity analysis three^f^ |  | Ref. |  | -1.7 (-8.2, 1.0) | 0.307 |  | 1.9 (0.2, 2.7) | 0.036 |
| Sensitivity analysis four^g^ |  | Ref. |  | 0 (-3.6, 1.7) | 0.99 |  | 1.6 (-0.7, 2.5) | 0.128 |
|  |  |  |  |  |  |  |  |  |

^a^ Estimated using Fine and Gray competing risk models with death as competing event.

^b^ Estimated using rank-based accelerated failure time models. Coefficients are [(1 – time ratios) × 100%], where time ratios are exp(β) coefficients.

^c^ Calculated as (1 – time ratio) × 3.13 years (the 3.13 years is the survival time for the no delirium dementia group from Kaplan-Meier plots with survival probability of 0.70).

^d^ Excluding participants who died or were admitted to a nursing home on the same episode as index or control episode.

^e^ Excluding the 10% least well-matched pairs based on Eucledian distance values.

^g^ Excluding participants who had their first diagnosis of delirium on the same day as their first diagnosis of dementia.
